# Meprin Metalloproteases Generate Biologically Active Soluble Interleukin-6 Receptor to Induce Trans-Signaling

**DOI:** 10.1038/srep44053

**Published:** 2017-03-09

**Authors:** Philipp Arnold, Inga Boll, Michelle Rothaug, Neele Schumacher, Frederike Schmidt, Rielana Wichert, Janna Schneppenheim, Juliane Lokau, Ute Pickhinke, Tomas Koudelka, Andreas Tholey, Björn Rabe, Jürgen Scheller, Ralph Lucius, Christoph Garbers, Stefan Rose-John, Christoph Becker-Pauly

**Affiliations:** 1 Institute of Anatomy, University of Kiel, 24118 Kiel, Germany; 2 Institute of Biochemistry, University of Kiel, 24118 Kiel, Germany; 3 Systematic Proteomics & Bioanalytics; Institute of Experimental Medicine; University of Kiel, 24105 Kiel, Germany; 4 Institute of Biochemistry and Molecular Biology II, Medical Faculty, Heinrich-Heine-University, 40225 Düsseldorf, Germany

## Abstract

Soluble Interleukin-6 receptor (sIL-6R) mediated trans-signaling is an important pro-inflammatory stimulus associated with pathological conditions, such as arthritis, neurodegeneration and inflammatory bowel disease. The sIL-6R is generated proteolytically from its membrane bound form and A Disintegrin And Metalloprotease (ADAM) 10 and 17 were shown to perform ectodomain shedding of the receptor *in vitro* and *in vivo*. However, under certain conditions not all sIL-6R could be assigned to ADAM10/17 activity. Here, we demonstrate that the IL-6R is a shedding substrate of soluble meprin α and membrane bound meprin β, resulting in bioactive sIL-6R that is capable of inducing IL-6 trans-signaling. We determined cleavage within the N-terminal part of the IL-6R stalk region, distinct from the cleavage site reported for ADAM10/17. Interestingly, meprin β can be shed from the cell surface by ADAM10/17 and the observation that soluble meprin β is not capable of shedding the IL-6R suggests a regulatory mechanism towards trans-signaling. Additionally, we observed a significant negative correlation of meprin β expression and IL-6R levels on human granulocytes, providing evidence for *in vivo* function of this proteolytic interaction.

The metalloproteases meprin α and meprin β belong to the astacin family of zinc endopeptidases and the metzincin superfamily. They are evolutionarily related to Matrix Metalloproteases (MMPs) and A Disintegrin And Metalloproteases (ADAMs)^1^, but possess unique structural and functional properties when compared to other metalloproteases^2^. Constitutive furin-mediated cleavage of meprin α along the secretory pathway results in the release of the enzyme into the extracellular space^3^ where it undergoes non-covalent oligomerization and can build huge complexes to form the largest secreted protease known to date^4^,^5^. In contrast, meprin β is found primarily tethered to the cell membrane but, under certain conditions, may be shed from the surface by ADAM10 or ADAM17^6^,^7^. Many transmembrane proteins have been identified as substrates for ADAMs including Notch, pro-inflammatory cytokines such as tumor necrosis factor-α (TNF-α) and its receptor TNFRI, adhesion molecules and the APP^8^,^9^,^10^,^11^,^12^,^13^,^14^,^15^,^16^. Interestingly, meprins were also shown to cleave ligands of the epidermal growth factor receptor (EGFR)^17^, as well as APP^18^,^19^ pointing to a partially shared substrate-repertoire of ADAMs and meprins.

Additionally, ADAM10/17 can cleave the membrane-bound interleukin-6 receptor (IL-6R)^20^,^21^,^22^,^23^,^24^, which plays a critical role in the initiation and propagation of inflammation^25^,^26^,^27^. The IL-6R is expressed in a restricted subset of cell types^28^,^29^,^30^,^31^ and can signal in two different manners, namely the predominantly pro-inflammatory trans-signaling via sIL-6R^32^ or the more regenerative classical signaling via membrane-bound IL-6R^33^,^34^.

There is evidence that highlight the potential of meprins to modulate the immune environment by cleaving mucin 2 important for intestinal mucus detachment^35^, processing bacterial proteins therby preventing invasion^36^, and modulating the activity of pro-inflammatory cytokines, such as interleukins (ILs), including IL-1β and IL-18^37^,^38^,^39^ as well as IL-6^40^, which are released in response to tissue injury and inflammation. This is further supported by distinct immunological phenotypes observed in meprin α and meprin β deficient mice^41^,^42^,^43^. For instance, meprin α knock-out animals showed more severe inflammation and intestinal injury in a DSS (dextran sodium sulfate)-induced colitis model compared to wild-type animals^44^, which is comparable to that observed in studies with significantly reduced expression of ADAM17 in mice^45^.

Until now, ADAM10 and ADAM17 are the only metalloproteases accredited with the ability to cleave the IL-6R^20^,^21^,^22^,^23^,^24^,^46^. However, the initial cleavage site identified by Müllberg and colleagues between Gln357 and Asp358^46^ with a negatively charged residue at the P1 prime (P1’) position does not coherent with predicted sequence preference for ADAMs that appear to favor more hydrophobic amino acids such as valine and leucine^47^. Indeed, recombinant ADAM17 cleaved an IL-6R peptide comprising a part of the stalk region, situated two residues N-terminal of QD between Pro355 and Val356^48^. This cleavage site was further substantiated by molecular modelling^49^. Interestingly, meprin α and meprin β were found to exhibit a striking preference for negatively charged amino acids at the P1’ position^50^, which correlates with the previously observed cleavage site in the IL-6R^46^. Here, we demonstrate that the IL-6R is indeed cleaved by soluble meprin α as well as membrane-bound meprin β. Ectodomain shedding of the IL-6R by meprins results in the generation of biologically active soluble IL-6R capable of inducing IL-6 trans-signaling.

## Results

### A peptide derived from the membrane proximal stalk region of the IL-6R is cleaved by meprins

**Figure 1:**
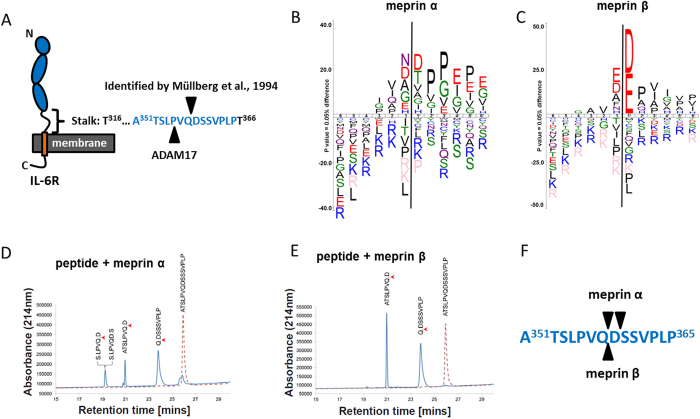
Meprin α and meprin β cleave a peptide of the IL-6R stalk region. (**A**) Schematic representation of the IL-6 receptor (IL-6R) in its membrane bound form. Indicated are the cleavage sites that were identified by Müllberg and colleagues^46^ and the one found for ADAM17 in a peptide cleavage assay^48^. (**B**) ICE logo representing the cleavage specificity of meprin α and (**C**) of meprin β both with a preference for negatively charged amino acids around the scissile bond. (**D**) Peptide cleavage assay analyzing part of the stalk region of the IL-6R comprising the previously identified cleavage sites as indicated in (**A**). HPLC and MALDI-TOF analyses revealed the cleavage site for meprin α and (**E**) for meprin β. (**F**) Summary of the cleavage sites identified for meprin α and meprin β. Note that both proteases cleave at the position (Q/D).

In 1994 Müllberg and colleagues identified a cleavage site within the stalk region of the IL-6R between Gln357 and Asp358 (Fig. 1A), which was assigned to a shedding protease’s activity, most likely ADAM17^46^. However, it has been demonstrated recently that negatively charged amino acid residues at the P1’ position are disliked by ADAM17^47^, but highly favoured by the metalloproteases meprin α and meprin β (Fig. 1B,C)^50^, which were further analyzed in our study. We therefore created a peptide spanning this particular region within the stalk of the IL-6R (Fig. 1A). The use of two different mass spectrometric technologies (LC-ESI MS and MALDI-MS) in parallel enabled identification of cleavage sites within the peptide (Fig. 1D,E). These results clearly showed that the peptide was cleaved, predominantly between amino acid residues glutamine and aspartate, after incubation with recombinant soluble meprin α or meprin β (Fig. 1F), with slightly faster kinetics of peptide digestion for meprin β (data not shown). These results prompted us to further analyze IL-6R cleavage by meprins in a cell culture model.

### Recombinant soluble ectodomain of meprin α but not meprin β can cleave the IL-6R in cultured cells

**Figure 2:**
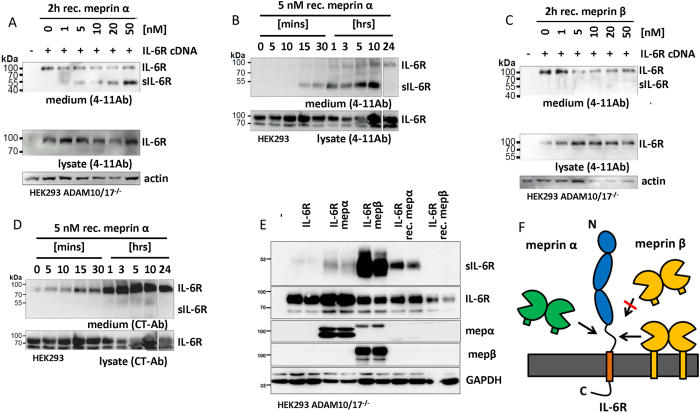
IL-6R cleavage by meprins generates soluble receptor independent of ADAM10/17. (**A**) Human IL-6R was overexpressed in ADAM10/17 dKO HEK cells. Forty-eight hours post transfection recombinant meprin α was applied to the cell culture. After 2 hours the supernatant was harvested and ConA precipitated. A clear increase in cleaved soluble IL-6R fragment of about 50 kDa was detected with increasing concentration of meprin α. Additionally, the full-length receptor was detected in the supernatant. (**B**) The transfected IL-6R was also cleaved by a rather low concentration of soluble meprin α (5 nM) in a time dependent manner. Again a probable full-length version of the IL-6R was detected. (**C**) For soluble meprin β no cleavage product of the IL-6R was detected in the cell supernatant. However, a signal for the IL-6R with a molecular weight of the full-length receptor was observed. (**D**) To further address secretion of the full-length IL-6R a C-terminal antibody (CT-AB) was used, detecting the cytoplasmic part. Here, the full-length version of the IL-6R was clearly detected in cell culture supernatants at approximately 100 kDA but not the cleavage product generated by meprin α. (**E**) Co-transfection of the IL-6R with meprin α or meprin β revealed cleavage products generated by both proteases. As a control, recombinant meprins were added. Only meprin α and not soluble meprin β produces sIL-6R. (**F**) Summarizing model indicating that only soluble meprin α membrane bound meprin β shed the IL-6R. Full-length blots are shown in Supplementary Information.

To avoid constitutive shedding of the IL-6R by ADAM proteases before meprin treatment as well as other protease-protease-interactions, IL-6R was transiently overexpressed in HEK cells deficient for ADAM10 and ADAM17^49^. Addition of active soluble recombinant meprin α led to a concentration (Fig. 2A) and time dependent (Fig. 2B) increase highlighted through the abundance of a cleavage fragment of the IL-6 R of approximately 50 kDa. Surprisingly, this was not the case after application of active recombinant soluble meprin β (Fig. 2C). Interestingly, we observed that transient transfection of IL-6R resulted in the release of a probable full-length form of the IL-6R of about 100 kDa into the supernatant (Fig. 2A–C). To verify this we used an antibody directed against the C-terminus of the IL-6R that exclusively detects the full-length IL-6R. Employing this antibody, we again analyzed cell culture supernatants from IL-6R transfected cells in the presence or absence of meprin α and indeed found the full-length version of the IL-6R in the supernatant which increased over time, whereas the meprin α generated fragment was not detected (Fig. 2D). To further evaluate the cleavage of the IL-6R by meprins we then transfected IL-6R overexpressing cells with full-length meprin α or meprin β. In these cells, not only meprin α but also membrane bound meprin β were both capable of cleaving the IL-6R to release a soluble fragment of about 50 kDa (Fig. 2E). Thus, we can state that soluble meprin α and membrane bound but not soluble meprin β cleave the IL-6R (Fig. 2F).

### Verification of the meprin cleavage site within the stalk region of the IL-6R

**Figure 3:**
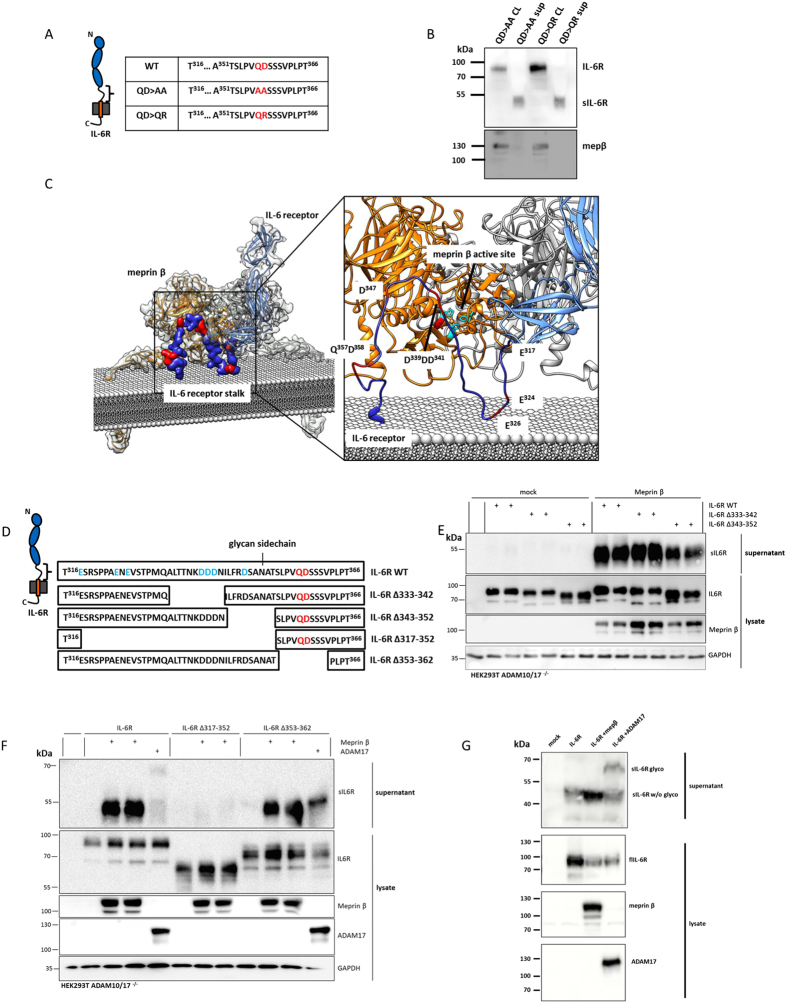
Determination of the meprin cleavage sites in the IL-6R. (**A**) Sequences of two QD (QD > AA and QD > QR) motif mutants that were anaylzed for meprin cleavage in ADAM10/17 dKO HEK. (**B**) IL-6R mutants QD > AA and QD > QR were co-transfected with meprin β. Surprisingly, sIL-6R was found in the supernatant 48 hours post-transfection, indicating that cleavage by meprin β does not occur between QD. (**C**) Structural analysis of the IL-6R (blue) fitted into the active site of membrane bound meprin β (orange/grey). It appears that type I transmembrane proteins, such as the IL-6R, require at least 20–25 amino acids in the stalk region to gain access to the active site of meprin β. The stalk of the IL-6R must form a loop to insert from the top of the active site cleft in cis-orientation that enables cleavage. Note the numerous negatively charged aspartate and glutamate residues that are present in the stalk (insert). (**D**) Amino acid sequences of four additional mutants that were analyzed, each with diverse parts of the stalk region absent. Note the N-glycan side chain that is present at Asn350. (**E**) Immunoblot analysis of the two IL-6R mutants Δ333–342 and Δ343–352 co-transfected with meprin β. (**F**) Immunoblot analysis of the two IL-6R mutants Δ317–352, missing most of the stalk region, and Δ353–362 missing the QD motive. For the Δ317–352 there was no cleavage fragment detectable after co-transfection with meprin β, whereas sIL-6R was seen in the supernatant of the Δ353–362 mutant upon co-expression with meprin β. This further indicates that the QD motif is not cleaved by meprin β. (**G**) As ADAM17 most likely cleaves in the region of the QD motif we co-expressed meprin β or ADAM17 together with the wild-type IL-6R and compared the molecular weights of the cleavage products. For meprin β a cleavage fragment was found at about 50 kDa, whereas ADAM17 generated a larger product of about 70 kDa. Thus it is highly likely that meprin β cleaves N-terminal and ADAM17 C-terminal of the glycan side chain, which accounts for the large difference in molecular weight. Full-length blots are shown in Supplementary Information.

To further demonstrate in a cellular system that the meprin α and meprin β mediated cleavage of the IL-6R takes place between Gln357 and Asp358, as observed in the peptide cleavage assay (Fig. 1), two mutants of the IL-6R were analyzed (Fig. 3A; QD in red) that were previously reported in^46^. The QD >QR mutant was analyzed, due to the fact that meprins strongly dislike positively charged amino acids around the scissile bond^51^. Surprisingly, both mutants were still shed after co-transfection with meprin β (Fig. 3B). Thus, we analyzed the structural properties of membrane bound meprin β based on the crystal structure of the ectodomain^52^ and fitted it with a model of the full-length IL-6R^53^ (Fig. 3C). We found that the orientation of the catalytic domain of meprin β requires a loop within the stalk region of the IL-6R of about 20–25 amino acids to obtain access to the active site cleft, where hydrolysis of a peptide bond occurs. It turned out that Gln357 and Asp358 are in too close proximity to the cell surface, which excludes cleavage of the IL-6R by meprin β at this particular site (Fig. 3C). Analysing the stalk region of the IL-6R revealed several other negatively charged glutamate and aspartate residues, which may be cleaved by meprin α and meprin β (Fig. 3C,D). To narrow down the cleavage site, four additional mutants of the IL-6R lacking various parts of the stalk region were analyzed (Fig. 3D). The first two deletion mutants, lacking amino acids Ala333-Asn342 and Ile343-Thr352 respectively (Fig. 3D), were still cleaved by co-transfected meprin β resulting in a single soluble fragment of the IL-6R (Fig. 3E). In the third mutant almost the entire stalk region spanning amino acids Glu317-Thr352, was deleted, except the last 14 residues including the QD motif. Here, no sIL-6R fragment could be detected in the supernatant upon co-transfection with meprin β indicating that shedding was completely abolished (Fig. 3F). These findings clearly exclude Gln357 and Asp358 as the cleavage site used by meprin β. To additionally validate this finding, ten amino acids were deleted including the QD motive (Ser353-Val362). This mutant was again shed by the co-expressed meprin β to release a fragment of about 50 kDa.

However, our mutation experiments indicate that the sIL-6R fragment generated by meprin β is smaller than the one produced by ADAM17. To further investigate this, we co-transfected ADAM10/17 double-deficient HEK cells with the IL-6R and meprin β or ADAM17. In the cell supernatants a smaller sIL-6R fragment was detected when meprin β was co-expressed (~50 kDa) compared to ADAM17 co-expression (~70 kDa) (Fig. 3G). Altogether this indicates a cleavage site N-terminal to the one used by ADAM17. We assume that the large difference in molecular weight between meprin and ADAM17 generated sIL-6R is due to the difference in length of the remaining stalk region and particularly due the absence or presence of glycosylated Asn350, respectively, as indicated in Fig. 3D^46^.

### Meprin generated sIL-6R is capable of trans-signaling

**Figure 4:**
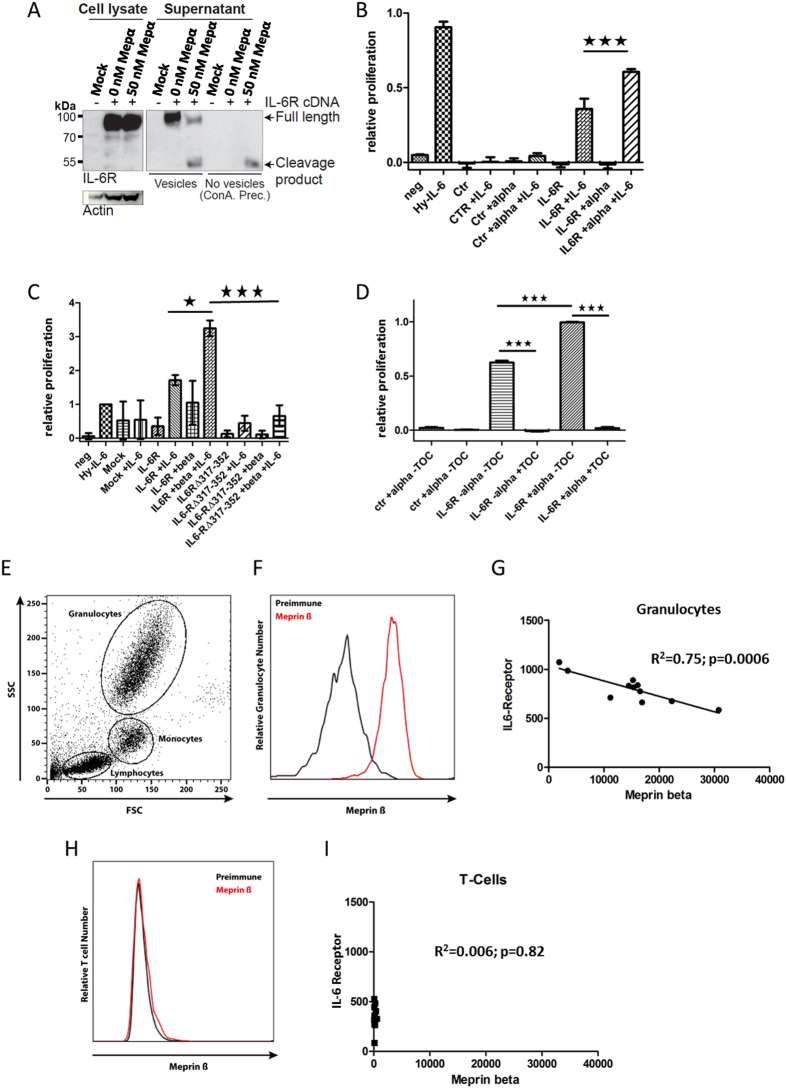
Functional aspects of meprin mediated IL-6R shedding. (**A**) To measure the biological activity of sIL-6R, we separated the meprin generated sIL-6R from the full-length form employing ultra-centrifugation. This resulted in a clean sample that only contains the sIL-6R. (**B**) Ba/F3-gp130 cell proliferation assay. The sIL-6R generated by meprin α revealed biological activity as indicated by the increase in cell proliferation in the presence of IL-6. Hy-IL-6, a fusion protein of IL-6 and the sIL-6R, was used as positive control. (**C**) As in (**B**) with sIL-6R generated by meprin β. (**D**) Same experimental set-up as in (**B**), additionally treated with Tocilizumab to block IL-6 signaling. (**E**) Scatter blott of a human blood sample analyzed by flow cytometry, demonstrating separation of granulocyte, monocyte and lymphocyte populations. (**E**) Meprin β specific antibody revealed expression on granulocytes, using pre-immune serum as control. (**F**) Eleven human blood samples from healthy donors were analyzed for meprin β and IL-6R levels on granulocytes. After linear regression a negative correlation between both proteins became evident. (**G**) On human T-cells there was no signal detected for meprin β, nevertheless there were differences in the amount of IL-6R. These are most likely due to ADAM10 cleavage. (**H**) As (**F**) but T-cells were analyzed. The linear regression showed no correlation.

We have shown that meprin α and meprin β proteolytically release sIL-6R from the cell surface. However, we also observed constitutive secretion of full-length IL-6R into the media, which was independent of meprins (Fig. 2A–C). This was proven by an antibody that recognizes an epitope within the intracellular C-terminus of IL-6R (CT-Ab) that only detects the upper band of about 100 kDa corresponding to the full-length receptor and not the proteolytically shed sIL-6R at 50 kDa (Fig. 2D). It is likely that this higher molecular weight form of the IL-6 R is secreted in microvesicles, similar to those observed in human serum^54^. Therefore, we employed ultracentrifugation to eliminate microvesicles^55^ that may contain full-length IL-6R from cell media that may interfere with functional assays of the sIL-6 R carried out in further experiments. After removal of microvesicles, only the cleaved form of the IL-6 R was detectable in cell culture supernatants (Fig. 4A). This purified fraction was used for functional assays in a murine B-cell line (Ba/F3)^56^ stably transfected with the signal transducing receptor gp130^57^,^58^. Cell culture supernatants of A10/17 dKO HEK cells were generated after transfection with the IL-6R and then either incubated with recombinant meprin α or co-transfected with meprin β. In both cases a significant increase in the proliferation rate of the Ba/F3-gp130 cells dependent on IL-6 was detectable (Fig. 4B,C), indicating that meprin α and meprin β produce bioactive sIL-6R. To proof that only meprin generated sIL-6R is mediating Ba/F3 cell proliferation, we additionally applied Tocilizumab, which blocks the IL-6 binding site of the α receptor, to conditioned media from meprin α treated HEK cells, overexpressing the IL-6R. Indeed, proliferation of Ba/F3-gp130 cells was completely abolished in the presence of the therapeutic (Fig. 4D).

### The IL-6R is cleaved on human granulocytes by endogenous meprin β

The IL-6R is highly expressed on immune cells of the human body^29^. Meprin β was shown to play a role during inflammatory processes, such as inflammatory bowel disease and nephritis, and therefore its expression on the surface of immune cells was suggested^59^,^60^. To analyze this with regard to IL-6R shedding, we took human blood samples from 11 healthy donors and analyzed the meprin β and IL-6R level on the cell surface by flow cytometry (Fig. 4E). We found a strong signal for meprin β on granulocytes that was clearly different from the pre-immune serum stained control (Fig. 4F). Interestingly, we observed a highly significant negative correlation between the meprin β and IL-6R surface levels. The more meprin β was detected the less IL-6R was present at the cell surface of granulocytes (Fig. 4G). This finding strongly points towards an *in vivo* cleavage of the IL-6R by meprin β under physiological conditions, at least on granulocytes. For T-cells, ADAM10/17 were described as the major sheddases of the IL-6R *in vivo*^61^,^62^. Therefore, we additionally analyzed these cells and could detect only very little meprin β at the cell surface, when compared to granulocytes (Fig. 4H). Regarding the IL-6R surface levels, however, there were differences seen between the 11 blood samples (Fig. 4G), which are in the same range as seen on granulocytes. However, these IL-6R levels did not correlate with the little amounts of meprin β detectable on T-cells, indicating other receptor shedding activities than that of meprin β (Fig. 4I).

## Discussion

**Figure 5:**
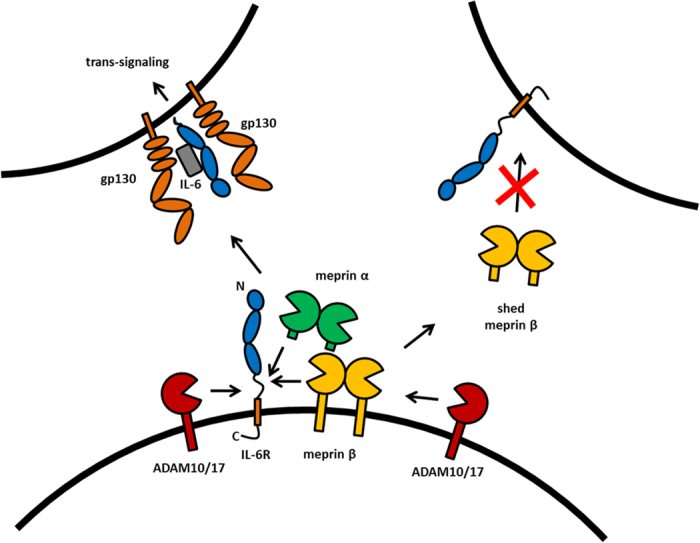
Regulation of IL-6R shedding by meprins and ADAMs. Soluble meprin α and membrane bound meprin β can generate sIL-6R. The cleavage however occurs at distinct sites when compared to ADAM10/17 mediated shedding. The sIL-6R can in all cases bind to IL-6 and gp130 on another cell and induce trans-signaling. Shedding of meprin β by ADAM10/17 might be a regulatory step to prevent generation of sIL-6R by membrane bound meprin β.

The IL-6R is an important signal transducer that can act via two mechanisms; classic signaling on the same cell and trans-signaling after proteolytic release acting on other cells that do not express the IL-6R^63^,^64^ (Fig. 5). Previously ADAM10 and ADAM17 were described to be the major shedding enzymes of the IL-6R^23^,^46^,^61^,^65^. However, some studies reported in literature regarding the cleavage site specificity of ADAM10/17 and the constitutive sIL-6R levels in serum gave rise to speculation of other proteases additionally involved in IL-6R shedding^47^,^54^.

Here, we describe meprin α and meprin β as two novel metalloproteases that are capable of shedding the IL-6R and thereby releasing biologically active sIL-6R fragments from the cell surface. We identified meprin α as the first soluble metalloprotease that can shed the IL-6R. Interestingly, meprin α was previously shown to cleave and release some ligands of the EGFR^17^, additionally demonstrating a partially overlapping substrate repertoire with ADAM10/17. On the other hand, meprin β membrane tethering appears to be a prerequisite for IL-6R shedding, because the soluble protease did not result in receptor cleavage. A similar observation was made for the APP where only membrane bound and not shed meprin β exhibits β-secretase activity^18^,^66^. This points towards strict regulation of meprin β activity at the cell surface as its shedding completely switches its preference towards certain substrates. Interestingly, ADAM10 and ADAM17 were previously identified as sheddases for meprin β^6^, indicating a complex scenario of cellular protease-protease-interactions and substrate cleavage competition in the regulation of the protease web^67^,^68^.

As the cleavage site within the IL-6R stalk region published by Müllberg and colleagues^46^ between Gln357 and Asp358 (QD motif) fits nicely to the specificity of both meprin metalloproteases^50^ and not to ADAM10/17^47^, we addressed cleavage of the IL-6R by meprins in detail. Surprisingly, we found that this particular QD motif is not used by meprin α and meprin β for the generation of sIL-6R. Instead, other negatively charged amino acids in the very N-terminal region of the IL-6R stalk are most likely cleaved by meprins supported by different IL-6R stalk deletion mutants. Additionally, the cleavage fragments of the IL-6R generated by meprins appeared to have a molecular weight approximately 20 kDa less than those released by ADAM10/17. This remarkable difference in size cannot only be explained by the different number of amino acids in the remaining stalk. This indicates cleavage by meprins N-terminal of the known N-glycosylation site (Asn350), which is still present in the ADAM10/17 mediated cleavage product^46^. Indeed, deglycosylation of ADAM17 and meprins generated sIL-6R revealed a shift in molecular weight towards a 35 kDa band (data not shown). Similar observations for ADAM17 mediated sIL-6R were previously published by Müllberg and colleagues^46^. We assume that the triple D-motif (D339DD341) and the two glutamate residues Glu324 and Glu326 are in a good distance to the membrane to be processed by meprin metalloproteases. Deduced from the crystal structure of meprin β^52^ and based on our model of the membrane bound enzyme in complex with the IL-6R, we conclude that the minimum number of amino acids adjacent to the membrane region is 20–25 that would allow correct orientation of the scissile bond within the active site cleft of meprin β. This explains why the QD motif is cleaved by meprin β only in the peptide assay and not in the full-length IL-6R as it is only eight amino acids apart from the membrane helix. Furthermore, cleavage of the IL-6R by ADAM10/17 between PV or QD, as well as the localization of cleavage sites in other known substrates^49^,^69^, suggests a structural conformation of these ADAMs where the catalytic domain is in very close proximity to the cell surface, as seen for meprin β, but with 180° rotation of the active site cleft.

We could show that the sIL-6R fragment that is produced by meprins has full bioactivity as demonstrated in the Ba/F3-gp130 cell proliferation assay^56^. This finding is in line with structural information on the IL-6R, IL-6 and gp130 complex as the IL-6R stalk is not involved in complex formation.

The *in vivo* function of ADAM10/17 mediated shedding of the IL-6R from activated immune cells, for instance T-cells and macrophages, has been demonstrated multiple times^70^. Nevertheless, IL-6R shedding under certain conditions cannot be clearly assigned to ADAM10/17 activity^54^. Thus, meprins may contribute to the release of sIL-6R under conditions where ADAMs do not play a major role. Indeed, we observed a significant correlation of meprin β and IL-6R on the surface of granulocytes, clearly showing that high expression of the protease is associated with low receptor levels. Thus, meprin β could determine the amount of IL-6R on granulocytes that is delivered to a site of inflammation and consequently may determine the amount of IL-6R that can be shed by ADAM proteases after immune cell activation. On the other hand, ADAM10/17 were suggested to be the relevant sheddase of the IL-6R on T-cells *in vivo*^61^,^62^. In line with this finding we did not observe a correlation between meprin β surface expression and the IL-6R on T-cells.

In summary, we describe two new sheddases for the IL-6R, namely meprin α and meprin β, which use a different cleavage site in the stalk region of the receptor, when compared to the well-known sheddases ADAM10 and ADAM17. Both meprins generate bioactive sIL-6R capable of trans-signaling. More importantly, we found a negative correlation between meprin β and IL-6R levels on the surface of human granulocytes (summarized in Fig. 5). Thus, the pro-inflammatory activity of meprin metalloproteases may be considered for the treatment of associated conditions, such as Kawasaki disease^71^ or inflammatory bowel disease^44^.

## Materials and Methods

### Materials and Chemicals

Reagents for molecular biology and protein standards were obtained from Fermentas (St. Leon-Rot, Germany). BCA protein assay kit and Western blotting reagents were from Pierce (Rockford, USA) and Amersham (Little Chalfont, United Kingdom). Complete^®^ protease inhibitor was purchased from Roche (Mannheim, Germany). Media for common cell culture was acquired from Thermo Fisher Scientific (Bremen, Germany). If not otherwise mentioned, all other chemicals were purchased from Sigma-Aldrich (Steinheim, Germany).

### Antibodies

Primary antibodies included rabbit-anti-CT-Ab (#SC661; Santa Cruz), rabbit-anti-actin (A2066, Sigma-Aldrich, Steinheim, Germany), rabbit-anti-GAPDH (14C10, Cell Signaling, Frankfurt am Main, Germany), rabbit-anti-meprin α (self-designed at Pineda, Berlin, Germany), and rabbit-anti-meprin β^72^, mouse-anti-human-IL-6R (4–11)^73^ and goat-anti-mouse-IL-6R (AF1830, R&D systems, Wiesbaden-Nordenstadt, Germany). Secondary antibodies used were Alexa Fluor 488, biotinylated antibodies (Molecular Probes, Eugene, USA) and peroxidase conjugated antibodies (Dianova, Hamburg, Germany).

### Recombinant protein and cell culture cleavage assays

Expression and purification of recombinant meprin α and -β was performed as previously described^4^,^74^. Briefly, proteins were heterologously expressed via baculovirus amplification in insect cells using the Bac-to-Bac system (Gibco, Thermo Fisher Scientific) according to the manufacturer’s instructions. Recombinant meprins were diluted in culture media devoid of fetal calf serum and subsequently incubation with cells at indicated concentrations for 2–24 hours.

### HPLC and Mass Spectrometry

344 μM synthetic IL6R peptide (ATSLPVQDSSSVPLP) was incubated with 5 nM recombinant meprin α or meprin β in 20 mM HEPES and incubated for 2 h at 37 °C. Reaction was stopped by addition of 0.1% trifluoroacetic acid (TFA) in ddH_2_O. Reverse high performance liquid chromatography (HPLC) analysis was performed using a gradient from 2% acetonitrile/0.1% TFA/H_2_O up to 60% acetonitrile/0.1% TFA/H_2_O in 38 minutes. All peak fractions were collected and analyzed by MALDI MS. Two hundred μL of each fraction collected by HPLC was concentrated in a vacuum centrifuge and redissolved in 20 μL of 3% acetonitrile, 0.1% trifluoroacetic acid. Samples were mixed with matrix (3 mg/mL α-cyano-4-hydroxycinnamic acid dissolved in 70% acetonitrile/0.1% trifluoroacetic acid) and spotted on a 384-spot target plate and measured in positive ion mode using an AB Sciex 5800 MALDI TOF/TOF MS (AB Sciex, Darmstadt, Germany). The matrix cluster (m/z of 877.034) was used for internal calibration of the MS spectra.

In addition, several fractions were analyzed by LC-ESI MS using a nano-HPLC system (UltiMate 3000, Dionex, Idstein, Germany) encompassing a trapping column (Acclaim PepMap 100; 100 Å pore and 5 μm particle size, 300 μm i.d. × 5 mm) and an analytical reversed-phase C18 column (PepMap; 100 Å pore and 3 μm particle size, 75 μm i.d. × 15 cm) that was coupled online to the mass spectrometer through a nanospray ion source (New Objective, Woburn, MA). Peptides were separated using a linear gradient at a flow rate of 300 nL/min. MS analysis was performed on an LTQ Orbitrap Velos mass spectrometer (Thermo Fisher Scientific, Bremen, Germany). Full MS scans (300–2000 m/z) were recorded at 60,000 mass resolutions. Following a full MS scan the five most intense signals were subjected to HCD fragmentation using a normalized collision energy of 40 and a dynamic exclusion of 5 s. Lock mass (445.120025) was used for data acquired in MS mode. MSMS spectra in both MALDI MS and LC-ESI MS were annotated manually.

### Knockout of ADAM10 and ADAM17 in HEK293T cells

Generation of CRISPR/Cas9 mediated knock-out of ADAM10/17 in HEK cells is described in^49^. Deficiency of ADAM10/17 was confirmed via activity assays, FACS and Western blotting.

### Cell culture

Cells were maintained in 5% CO_2_ atmosphere at 37 °C in Dulbecco’s Modified Eagle’s Medium (Thermo Fischer, Darmstadt Germany) supplemented with 10% (v/v) Fetal bovine serum (FCS) as well as penicillin/streptomycin (Thermo Fischer, Darmstadt Germany). For treatment of cells with recombinant protein, 48 hours post transfection, cells were cultured without FCS prior to analysis due to the meprin-inhibiting properties serum^75^. For co-transfection experiments cells were cultured 24 hours without FCS.

### Cell viability assay

The biological activity of soluble IL-6R was analyzed as described previously^76^. In brief, equal amounts of Ba/F3-gp130 cells were incubated with 50% conditioned cell culture supernatant and/or recombinant proteins as indicated in a final volume of 100 μl per well in a 96 well plate. Cell viability was determined using the Cell Titer Blue Cell viability assay reagent (Promega, Karlsruhe, Germany) according to the manufacturer’s instructions. To block IL-6 signaling Tocilizumab was added at a concentration of 10 μg/ml.

### Expression Plasmids

Expression plasmids containing the cDNA encoding the human IL-6R and IL-6R variants with deletions within the stalk region have been described previously^77^.

### Flow cytometric analysis

Surface expression of meprin β and IL-6R was analyzed on human blood cells. Therefore human heparinized whole blood samples were blocked with Fc block (Human TruStain FcX, BioLegend) diluted 1:100 in FACS buffer (1% BSA [w/vol] in PBS) for 15 minutes and subsequently stained with either anti-meprin β (hEcto1, polyclonal rabbit against human meprin β ectodomain, Pineda) or the pre-immune serum as a negative control, diluted 1:500 in FACS buffer for 1 hour, followed by 30 minutes incubation with the secondary antibody, goat anti-rabbit Alexa Fluor 488 conjugate (Life Technologies), diluted 1:300. For detection of IL-6R and CD3, cells were stained with either anti-IL6R-APC (clone UV4, BioLegend) or the corresponding isotype control (mouse IgG1, κ BioLegend) or anti-CD3-FITC (clone UCHT1, Biolegend) diluted 1:100. All incubations were carried out at 4 °C. Lysis of erythrocytes was performed in RBC Lysis/Fixation Solution (BioLegend) subsequent to staining procedure. Labeled cells were analyzed utilizing a FACS Canto II flow cytometer (BD Biosciences) and data were evaluated with FlowJo (Tree Star) software. T cells were defined as CD3 + whereas granulocytes were gated by FSC/SSC as SSChigh.

All individuals underwent a written, informed-consent process approved by the ethics commission of the Medical Faculty of Kiel University (A 102/14).

### Protein lysis, ultracentrifugation, immunoprecipitation, acid precipitation and immunoblotting

After removal of supernatant media, cells were scraped in ice-cold PBS, before centrifugation at 5,000 rpm for 10 minutes. Supernatants were cleared via centrifugation at 5,000 rpm for 10 minutes before Concanavalin A (ConA) immunoprecipitation or acid precipitation. To exclude the full-length receptor on microvesicles in the supernatant, serial centrifugation at 4,000 rpm for 3 minutes, at 13,000 rpm for 15 minutes and ultracentrifugation at 55,000 rpm for 2 hours was performed. Cells were lysed in PBS containing 1% triton-X with complete^®^ protease inhibitor. Fifty μl of (ConA) beads (GE Healthcare, Freiburg, Germany) was washed once in PBS and added to 1 ml of media for precipitation of all glycosylated proteins. The bead/media mixture was incubated overnight at 4°C before centrifugation at 5,000 rpm for 5 minutes. The supernatant was discarded and the pellet heated to 95°C for 10 minutes in 3 x Laemmli buffer^78^ before loading on an SDS-PAGE gel.

Trichloroacetic acid (TCA) precipitation of soluble proteins was carried out as follows. Centrifuged media (1 ml) was mixed in a 1:1 ratio with 20% TCA solution w/v before incubation on ice for 30 minutes, then centrifuged at 14,000 rpm for 20 minutes at 4 °C. The pellet was subsequently washed with 350 μl acetone. After inverting several times, the pellet was centrifuged for 15 minutes at 14,000 rpm at 4 °C. The supernatant was decanted off and the pellet allowed to dry overnight at room temperature. Resuspension of the pellet via 20 μl 3 x Laemmli buffer was achieved by repeated pipetting and heating the sample to 95 °C for 10 minutes, which was followed by SDS-PAGE and immunoblot analysis.

### Statistical analysis

All values are expressed as the mean ± SEM. Difference among mean values were analyzed via one-way ANOVA followed by Tukey post-Hoc test or the linear regression was analyzed using GraphPad Prism 5. In all analyses the null hypothesis was rejected at p < 0.05.

### Molecular modelling

Molecular modeling was performed using Modeller^79^. As a basis the meprin β and APP structural model was used^52^. All molecular imaging was performed using UCSF Chimera^80^. All methods were performed in accordance with the relevant guidelines and regulations of the University of Kiel.

## Additional Information

**How to cite this article:** Arnold, P. *et al*. Meprin Metalloproteases Generate Biologically Active Soluble Interleukin-6 Receptor to Induce Trans-Signaling. *Sci. Rep.* **7**, 44053; doi: 10.1038/srep44053 (2017).

**Publisher's note:** Springer Nature remains neutral with regard to jurisdictional claims in published maps and institutional affiliations.

## Supplementary Material

Supplementary Information
