# Supplementary material for: Meprin Metalloproteases Generate Biologically Active Soluble Interleukin-6 Receptor to Induce Trans-Signaling
Source: Sci Rep. 2017 Mar 9;7:44053. doi: 10.1038/srep44053 (PMC5343444; doi:10.1038/srep44053)

Supplementary Information

Full-length Western blots are shown in the same order as they appear in the main manuscript.

Fig2A

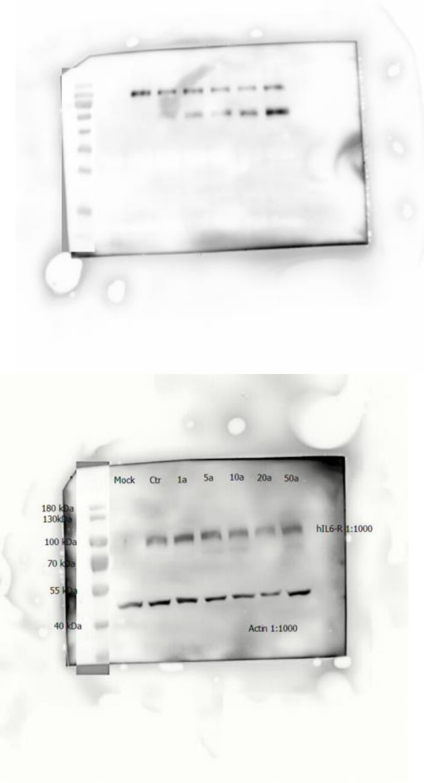

Fig2B

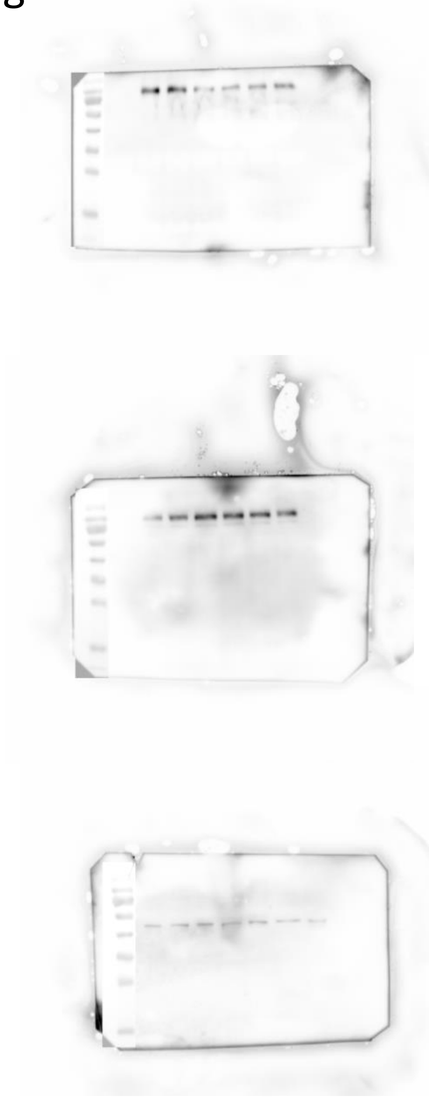

Fig2C

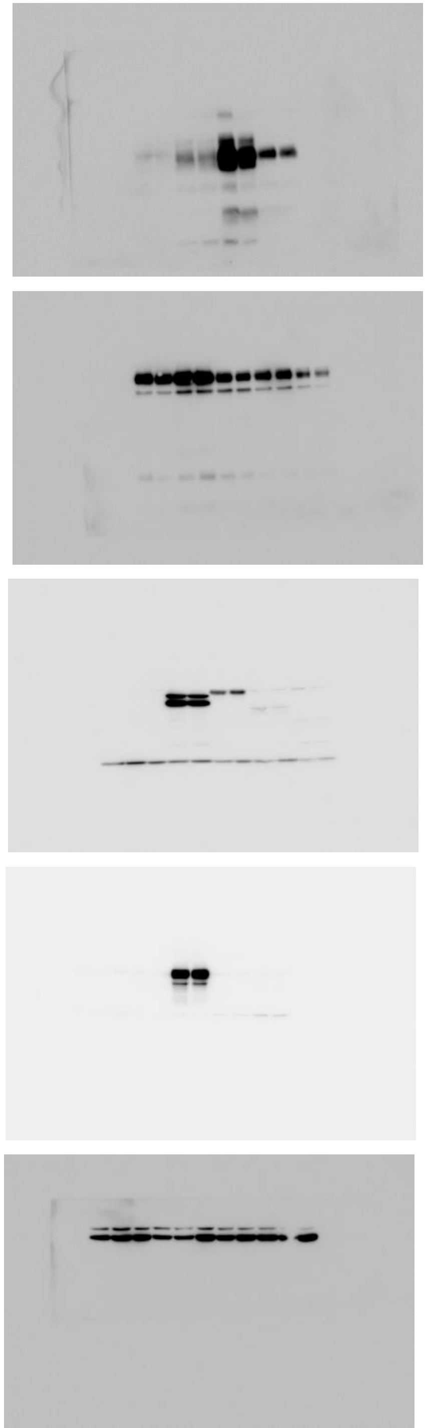

Fig3B

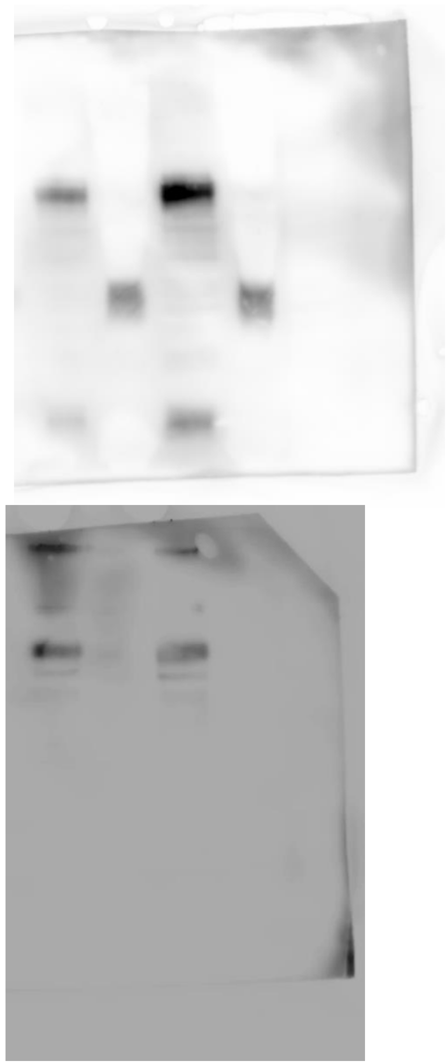

Fig3E

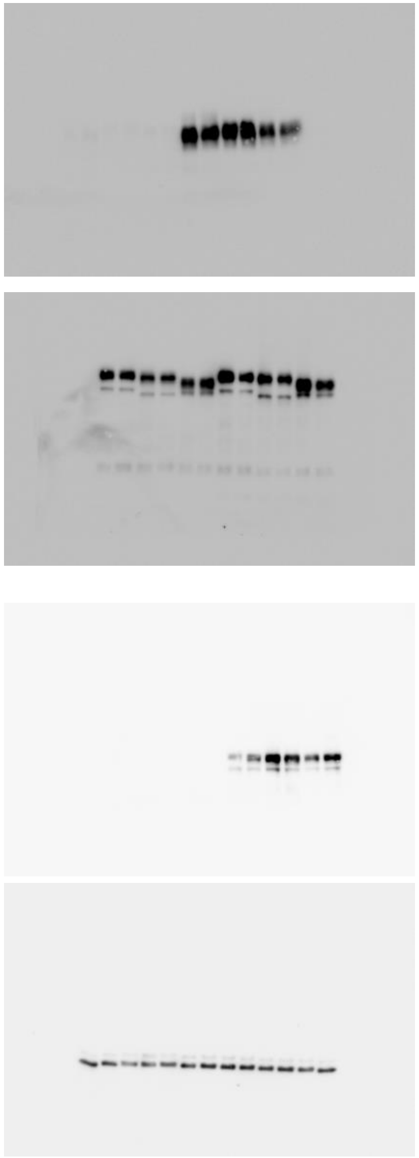

Fig3F

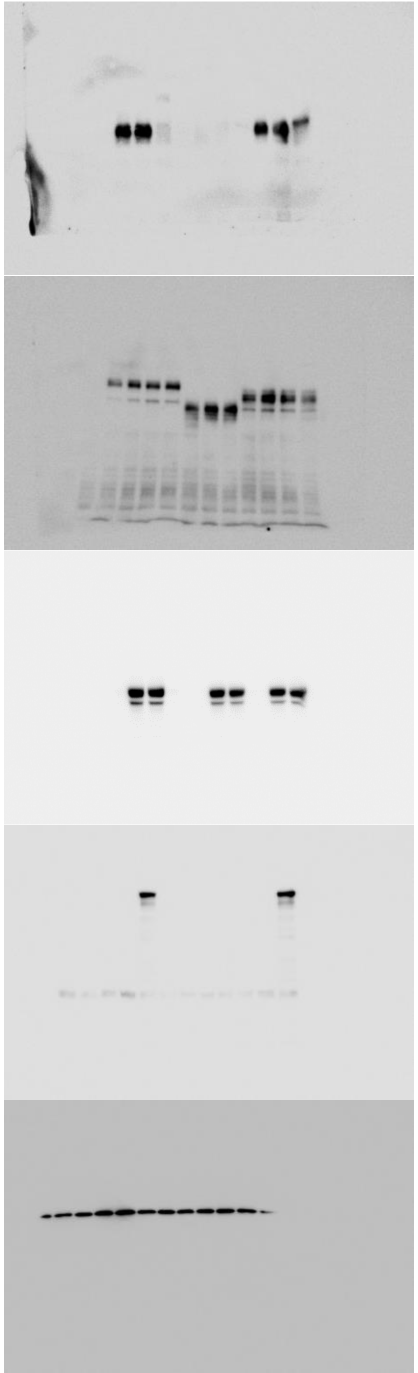

Fig3G

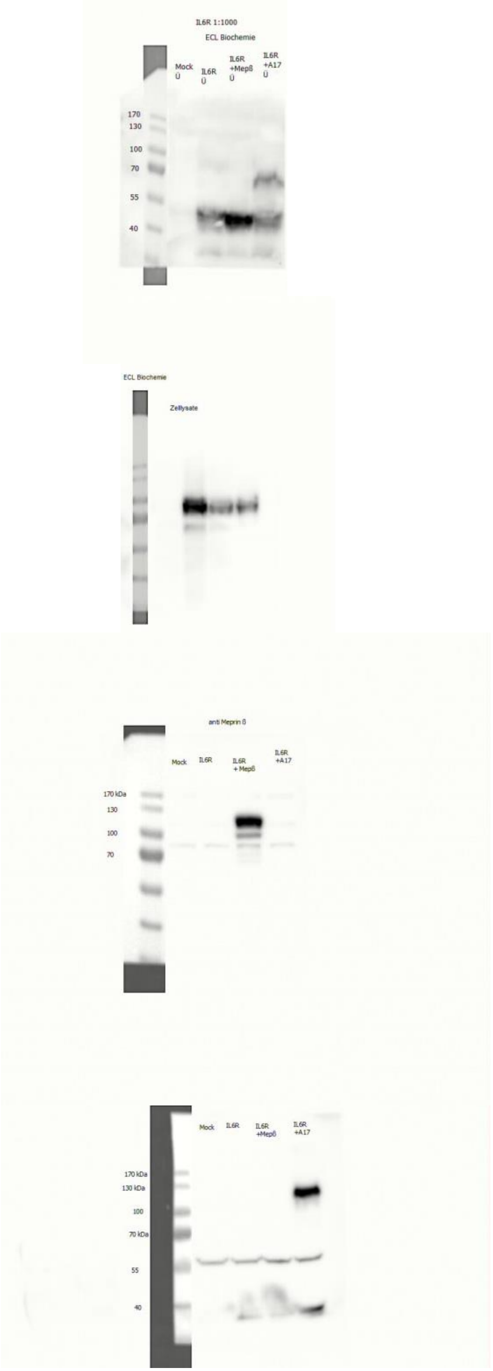

Supplement: Supplementary Information [file srep44053-s1.pdf]
